# Food for thought: an exploratory study of how physicians experience poor workplace nutrition

**DOI:** 10.1186/1475-2891-10-18

**Published:** 2011-02-18

**Authors:** Jane B Lemaire, Jean E Wallace, Kelly Dinsmore, Delia Roberts

**Affiliations:** 1 Department of Medicine, Faculty of Medicine, University of Calgary, Health Sciences Center, 3330 University Drive NW, Calgary, AB, T2N 4N1, Canada; 2 Department of Sociology, Faculty of Social Sciences, University of Calgary, 2500 University Drive NW, Calgary, Alberta, T2N 1N4, Canada; 3 Faculty of Kinesiology, University of Calgary, 2500 University Drive NW, Calgary, Alberta, T2N 1N4, Canada; 4 Selkirk College, 301 Frank Beinder Way, Castlegar, British Columbia, V1N 4L3, Canada

## Abstract

**Background:**

Nutrition is often a casualty of the busy work day for physicians. We aimed to explore physicians' views of their nutrition in the workplace including their perceptions of the impact of inadequate nutrition upon their personal wellness and their professional performance.

**Methods:**

This is a qualitative study of a sample of 20 physicians practicing in a large urban teaching hospital. Semi-structured open ended interviews were conducted to explore physicians' views of workplace nutrition. The same physicians had agreed to participate in a related nutrition based wellness intervention study that compared nutritional intake and cognitive function during a day of usual nutrition patterns against another day with scheduled nutrition breaks. A second set of interviews was conducted after the intervention study to explore how participation in the intervention impacted these views. Detailed interview content notes were transcribed and analyzed independently with differences reconciled by discussion.

**Results:**

At initial interview, participants reported difficulty accessing adequate nutrition at work, linking this deficit with emotional (irritable and frustrated), physical (tired and hungry), and cognitive (difficulty concentrating and poor decision making) symptoms. In addition to identifying practical barriers such as lack of time to stop and eat, inconvenient access to food and poor food choices, the physicians described how their sense of professionalism and work ethic also hinder their work nutrition practices. After participating in the intervention, most physicians reported heightened awareness of their nutrition patterns and intentions to improve their workplace nutrition.

**Conclusions:**

Physicians report that inadequate workplace nutrition has a significant negative impact on their personal wellness and professional performance. Given this threat to health care delivery, health care organizations and the medical profession need to address both the practical and professional barriers identified.

## Background

There is growing empirical evidence of an association between physicians' wellness and their ability to deliver quality health care [1-9] and yet physicians are often unable to tend to their wellness [10]. Physicians report that they frequently cannot eat and drink properly or at all during work hours [11-14]. Research has also shown an association between workplace nutrition and cognition for physicians [15]. The primary objective of our study was to explore hospital based physicians' views of how they experience poor nutrition in the workplace. Specifically, we asked about whether or not they have difficulty meeting their nutritional needs at work, their perceptions of the personal and professional impact of suboptimal nutrition, and the contributing barriers. The secondary objective was to further explore the same physicians' views and attitudes towards workplace nutrition after they had participated in a related study involving a nutrition-based intervention [15].

## Methods

During May and June of 2008, we interviewed a volunteer sample of 20 staff physicians from various medical specialties who were recruited from the doctors' lounge of a large urban teaching hospital. The average age of participants was 47 (range = 36 to 64 years), and 3 were women. Their average BMI was 25 kg/m2 (range = 20 - 38), all were non-smokers and most (75%) exercised at moderate to high intensity for 30 minutes or longer for at least 2 to 4 days per week. They represented a variety of medical practice areas where 10 participants were from a medical specialty (e.g., General Internal Medicine, Neurology, Intensive Care Medicine), 8 from a surgical specialty (e.g., General Surgery, Plastic Surgery, Otolaryngology), and 2 were from primary care (e.g., Family Medicine, Hospitalist). All had also agreed to participate in a related study involving a nutrition based wellness intervention [15]. The physicians were first interviewed, prior to participating in the intervention study, to explore their views of how they experience poor nutrition in the workplace, and again, after their participation in the intervention, to explore how their participation impacted these views. No physicians dropped out of the study.

The related intervention study [15] assessed hospital physicians' nutritional intake, cognitive function and hypoglycemic symptoms during two similar work days: one where they followed their usual eating and drinking habits and another where they were provided with nutritious food and drink at enforced scheduled intervals. The intervention was designed to ensure that physicians consumed nutrients and fluids at regular intervals throughout their work day and had four key elements: providing healthy nutrition choices; enforcing nutrition breaks; maximizing ease of accessibility; and offering cost free nutrition. Nutrition provided during the study period on the intervention day was based on the recommendations of Canada's Food Guide. On average, food and beverages were provided in six small meals. This varied according to the number of hours worked by each participant and their individual nutritional choices. At each scheduled nutrition break, the research team contacted participants through hospital paging. Ready-to-consume and cost free nutrition was either waiting for physicians at the centrally located doctors' lounge or was brought to their practice location. On both of the study days, cognitive function, nutrient/fluid intake, urinary output, frequency of hypoglycemic symptoms and capillary blood glucose were measured by the research coordinator every two hours. Analysis of these data showed a higher nutrient intake and improved hydration for the study participants on intervention versus baseline days. The intervention was associated with significantly superior scores on cognitive function testing, and lower and less variable blood glucose levels. Although not statistically significant, there was also a trend toward the reporting of fewer hypoglycemic symptoms. The participants were blinded to these results during the intervention study and also at the time of the second interview exploring the impact of the intervention on their views of workplace nutrition.

The interviews were conducted by the first author, a female internal medicine consultant, clinical professor of medicine, and colleague of the participants. The interviewer also holds an official appointment as Vice Chair, Physician Wellness and Vitality, Department of Medicine and has prior multidisciplinary research experience. The interviews took place at the hospital, in a private setting and lasted between 15 and 45 minutes. The interview questions analyzed in this paper are outlined in the results section below and were embedded in a more detailed set of questions that served as the entry and exit interviews for the nutrition based wellness intervention studying the association between workplace nutrition and cognition. The questions were guided by previous data from local research where both qualitative and quantitative data repeatedly identified poor workplace nutrition as a wellness concern for physicians [13,14]. In general, the initial questions required a yes/no response in order to determine what proportion of the physicians reported certain nutritional experiences. These were followed by open ended probes intended to elicit specific examples of how the physicians experience poor workplace nutrition. In addition, prompts were used in order to further explore the question, again followed by open ended probes framed to elicit the physicians' personal experiences.

The questions were not pilot tested, and no interviews were repeated for the same content. The detailed interview notes were transcribed into a typed summary and independently reviewed by the two lead co-investigators using an inductive strategy through open and selective coding to derive the predominant themes. Given the exploratory nature of the research, the interviews were completed with all 20 participants even if data saturation occurred on occasion with some of the major themes. This strategy also produced a richness of illustrative examples. Interview transcripts were not returned to participants for comments or corrections. All, however, were sent a summary report of the analyzed interview data and asked for comments, and no negative feedback related to accuracy or interpretation was received. Ethics approval for this study was obtained from the Conjoint Health Ethics Review Board of the University of Calgary.

## Results

During the first interview, the physicians were asked questions aimed at exploring their perceptions of workplace nutrition. We inquired as to how they experience inadequate nutrition in the workplace in terms of their personal wellness and professional performance, and whether or not they perceived a link between their nutrition and cognitive performance. They were also asked about what they viewed as the potential barriers to nutrition in the workplace. After completion of the intervention study described above, the participants were interviewed again to explore whether or not their participation had influenced their awareness of their work nutrition patterns and their intention to modify their future nutrition practices. The following themes emerged (Table 1).

**Table 1:** Physicians' views of their workplace nutrition

Impact of inadequate nutrition	
Emotional symptoms	Irritability, impatience, frustration, emotionally drained

Physical symptoms	Tired, hungry, nauseated, feel unwell, hypoglycemic symptoms

Cognitive symptoms	Can't focus, concentrate or think clearly, poor/slower decision making and performance, less efficiency

Negative impact on ability to complete work	Decreased efficiency, lack of focus

Negative impact on interactions with colleagues, other health care professionals and patients	Irritability, less gregarious

**Barriers to nutrition**	

Lack of time	Too busy, no time to eat, heavy workload, timing of work schedules

Limited access to nutrition and water and/or inconvenience	Location of food stations, hours of operation, line ups, no areas to store food from home

Limited food choices	In terms of quality, healthy foods, appeal, variety

Work ethic	Need to get the work done, work and patients come first

Professionalism/doctors attitudes	Inappropriateness of eating around patients, carrying food around, physician does not prioritize personal wellness

Cost	

**Impact of participating in a nutrition based wellness initiative**	

Increased awareness of workplace nutrition and impact	Noted their own irregular or poor eating and drinking habits, increased awareness of link between nutrition patterns and mood and performance

Intention to change future nutrition habits	Will snack and drink more regularly, will not skip meals

### Impact of Inadequate Nutrition

We asked the physicians "*Do you sometimes have difficulty eating and drinking during work hours?*" and all answered yes. We then asked *"Think back to a busy work day where you maybe did not have time to eat and drink properly. Do you think it had an impact on you? In what way? How did you feel physically, psychologically, cognitively? Do you think it had an impact on your work? In what way? Do you think it had an impact on how you treated your colleagues, other health care professionals? Did it impact your ability to complete your work?" *All of the participants reported that they thought that inadequate nutrition had some impact on them and almost all reported experiencing emotional symptoms including irritability, frustration, decreased patience and feeling emotionally drained. Most reported they have felt physical symptoms such as fatigue, hypoglycemic symptoms (shaky, tremors, sweats, headache, lightheaded, hunger, nausea), and general malaise. Many also described a perceived impact on their cognition, citing symptoms such as difficulty concentrating, lack of focus, inability to think clearly, poor or slow decision making, and generalized inefficiency. Many study participants admitted that they felt the impact of inadequate nutrition at times led to an inability to complete their work, and they offered examples such as decreased efficiency, lack of focus, less willingness to discuss patient care issues with colleagues and having to be much more deliberate and methodical when hungry. Examples of negative interpersonal interactions included being less patient and less gregarious. The following quotations illustrate some examples of the impact of inadequate nutrition experienced by physicians.

I think you can be distracted, irritable, have poor interactions if hungry enough. Your agenda changes and you just want to get the hell out of here. Hopefully, you don't give in to it. People get irritated and emotionally tapped out and don't want to think through the problem. (#20)

If I eat breakfast, I need lunch or I feel diaphoretic and shaky. So I cut out breakfast years ago because I can't get regular lunch. So if I miss meals irregularly it becomes an issue. I'm not hungry at lunch, I have no cravings, I have trained my body to miss lunch. If I deviate from my routine, yes, otherwise, no impact [of poor nutrition]. I feel shaky, get sweats, have decreased concentration, lack of focus, things don't seem to go right. I need fine motor skills during surgery and I will note a little bit of a tremor that almost makes the case impossible to do. Then I get frustrated and it makes things worse. (#12)

My attention is poor and I think about food and drink, which is a distraction. I get headaches, feel fatigue, less motivated and irritable. I'm not aware that it impacts my interactions with colleagues and my ability to complete work, but overall, it [hunger] may decrease efficiency. (#9)

[Impact of poor nutrition on work] Probably, but I would like to think not! [Impact on how he treats colleagues] I don't know, it's hard to say, hard to compare to what you could do best on that day. Maybe it takes you longer and you have to focus more. [Ability to complete work] Probably. At night, when you get older, it's harder. I have to focus on the real meat of it [work] and feel massive fatigue, especially if I haven't eaten. (#14)

In order to further explore the link between nutrition and cognition, we next asked the participants *"Do you think there is an association between a physician's nutritional intake during work hours and their cognitive function? Why or why not? What do you think the important consequences might be?" *Most participants felt strongly that there was an association between their nutrition and cognition. The physicians who either did not think there was an association or were not sure, often went on to qualify their opinion by adding comments suggesting they recognize within themselves the potential for lack of acknowledgment of this linkage. The following quotations illustrate some examples of the physicians' perceptions of the link between nutrition and cognition, and the conflict voiced by the physicians.

Absolutely [link between nutrition and cognition]. I know that myself. When I feel hungry, I can't focus. I get slower around 4 am when I am hungry and tired and not drinking enough water, not peeing. Consequences for patient care? For sure! (#1)

Absolutely! If I grab food on the run it doesn't feel as well as a rest and a read of the newspaper or a visit or a curbside consult [with colleagues]. Rest and food also equal socialization. Consequences [of good nutrition] are performance and less stress. I am thinking clearly, and am more efficient and organized. (#2)

I don't believe so [association between nutrition and cognition]. You have to be excessively hypoglycemic before cognitive impairment, or I hope that is true! I do feel sleepy after the mid day meal on the Monday. At times I am short tempered with staff and surly and sometimes they will bring me food and it helps. Does it affect how I treat colleagues? Yes, from an emotional and interpersonal way, but heaven forbid it would impact patient care. [Ability to complete work]I don't think so. I would notice it and eat. (#8)

I don't notice it in myself [association between nutrition and cognition]. I feel hungry but I don't think I "bonk". But perhaps I am lacking insight. I feel I perform well all the time although I do notice if I get on my bike to ride home and haven't eaten well, then I will bonk. (#11)

Suboptimal nutrition and restfulness should impact on cognition. Maybe it is surgical arrogance that makes me think not. To me, nutrition and sleep deprivation are linked. I am more likely to do a procedure not as slickly if it is routine, run of the mill, by rote, but when it is super technical, it is way easier to get switched on. For example, last week, the surgery took 6 hours, went fine, no issues, went well. When I was writing the discharge prescription at the end of the case, I made a prescription error, when the hard part was over. There is also a narrow view of "standard of care." How about our role of communicator, and educator? What if the patient has a ton of questions, and I put them aside because of fatigue and hunger? (#17)

### Barriers to Adequate Nutrition

We asked the physicians *"Do you think that you and other doctors are not always able to ensure adequate nutrition during work hours? If so, what barriers make it difficult for you to eat and drink in a healthy way during work hours?" *The major theme that emerged was that of lack of time. Doctors stated they are just too busy to even think about eating, that there is no time during their work day to stop and eat, that their workload prevents them from taking time to eat, and that their work schedules (e.g. business meetings or meetings with patients' families over the lunch hour, operating room cases or clinics running through lunch) make it difficult to access nutrition on a regular basis. The second major theme was limited or inconvenient access to nutrition during the physicians' work day. They described how the physical spaces within the hospital hinder their access to food, including having to walk long distances from the unit where they work to the food areas, waiting for elevators, and waiting in long line ups once they get to the food stations. In addition, they noted that current limited after-hours access to food is impractical given that the hospital staff work 24 hours per day. The participants also described inadequate food storage areas for items they bring from home. Many participants identified food choices as a major barrier, describing poor quality food, restricted healthy choices, and limited variety, while very few identified cost of healthy food as a barrier.

In addition to these practical access issues, the physicians also identified issues that reflect the culture of medicine and how physicians' professionalism deters them from taking care of their workplace nutrition needs. For example, many physicians reported that work and their patients come first, that they feel compelled to "just get the work done", and that taking time for nutrition delays caring for ill patients. Several participants also described profession related barriers, including the medical profession's low prioritization of workplace nutrition for physicians in general and a feeling of awkwardness or unprofessionalism when either carrying food around in the workplace or eating in patient care settings. The following quotes illustrate examples of some of the practical and professionalism barriers that participants identified.

I'm too busy to give it [nutrition] the thought it needs. It's hard to carry food around and I think I lack interest in nutrition. (#11)

It's not an access issue, the quality [of food] sucks. I would pay more if there was better food. I like to sit down to eat. Eating interrupts the work day and lengthens it. I am stubborn. Work stuff comes first. I cannot be non productive or inefficient. (#8)

I can't bear the food. The doctors' lounge [food] is OK. Bringing in three meals is too much hassle and choices for healthy foods are limited. I can't stand in line for food. There is no time with urgency of cases and the workload. (#4)

Barriers: Time is number one; too many pressing things that need to be done right now. There are awkward social pressures also, I feel awkward eating in the clinic. There is no microwave, I don't want to carry forks and knives, and I don't want to walk past a bank of patients with food, I feel self conscious. So I am trapped in the [clinic] eating cookies and chocolate and coffee and creamers and juice, no access to healthy foods, and technically am eating patient food. Club soda and orange juice is restorative because it reminds me of being home on my porch watching the dog drinking soda lime. (#19)

Barriers are time, the pressure of getting things done during the day that you can't put off, and poor availability [of food] especially in the evening and nights, when not a lot is available. If I am in the hospital all night, I try to eat early otherwise I can't get proper food. What is available? Chicken fingers and fries. Now at least the sandwich bar is open later and occasionally I can get a veggie burger or salmon burger. During the day reasonable food is available but I can't get to it. (#14)

The operating room is a food wasteland. There used to be food on the 7^th ^floor [operating room area] but now there are vending machines. It makes it difficult to get hydration and caloric intake. Same for the anesthesiologists. There is a lot of pressure in the OR. We need to be conscientious about intake. And there is no readily available drinking water. The social trend that I abhor is the lack of clean drinking water. No drinking fountains and need to buy a three dollar bottle of [brand] water that is an environmental nightmare. They removed juices from the operating room. In a pinch, the nurses will try to give you a drink and they might get the straw up your nose! (#13)

### Impact of Participating in a Nutrition Based Wellness Initiative

We asked the physicians, *"Has your participation increased your awareness of your nutrition patterns during your work day? If yes, how so?" *Almost all of the physicians responded that their participation in the initiative has increased their awareness of their own nutrition patterns, describing how they became more aware of their irregular or poor eating and drinking habits, and/or noted increased awareness through reinforcement and improved knowledge about nutrition. Some physicians reported an increased awareness of the link between their nutrition patterns and their mood and performance. The physicians who did not think that participation in the initiative had increased their awareness of their nutrition patterns reported that they already knew they had good or bad nutritional habits.

We also asked the physicians, *"Has your participation influenced how you will eat and drink during your work day? If yes, how so?" *The majority of the physicians reported that their future nutrition patterns will be influenced, commenting that they will snack and drink more regularly while at work, make an effort to prepare snacks to bring to the hospital, and be more careful about their eating habits such as having breakfast daily and not skipping other meals. Several participants felt they will not change their nutrition patterns. Reasons stated included affirmation that they will continue their current patterns despite knowing they should change them for the better or that their current eating habits are effective. Some physicians were ambivalent, and suggested that they might change their nutrition patterns if food was more readily available and accessible, or if they had more information on healthy nutrition. The following quotes illustrate examples of some of the physicians' views after participation in the nutrition based wellness intervention study.

First, it [the intervention] outlined the importance of breakfast. It makes you feel better. Second, the whole notion of spacing meals, a little here and a little there, fewer calories at a time, so there is no catch-up phenomenon. It adds up to fewer calories than my usual smorgasbord late in the day. (#7)

For sure! I just became really aware of how hungry I really was and that hunger would add to my sarcasm, I would get glib [with patients]. The irritability, getting spaced out without food is improved. I bought a water bottle and cut back on coffee. I still bring food from home and feeling better on the second day of the study added to my work happiness. (#19)

I recognize that when I get yawny or tired, it is because of low blood sugar. Then I like to run up and get something to eat or drink. Being in the study has reinforced the snack thing. Now I bring 2 fruit and trail mix. I don't get as many symptoms and am not as tired when I eat snacks. I come regularly up to the doctors' lounge or elsewhere to get snacks. Before, it was intermittent. I pace myself now to assure regular eating. I come for lunch regularly now, before it was intermittent. The nutrition issues were brought to my attention by the study, otherwise I would not have changed my habits. (#5)

I think I will be more careful about eating regularly. Funnily enough, I have been more careful the last couple of years because I can't do it like I used to. I don't have the same stamina or the same focus for hours on end. It's the same with food; if I do miss it it's harder. (#14)

Before I tended to not remember when I had eaten. Now maybe I can better correlate mood and performance. Because of the hypoglycemic questionnaire I started to realize I had symptoms of that even before I knew it. I am trying not to skip meals, and trying to snack, and to prepare food for work ahead of time. (#9)

I've started to hunt down trail mix so I can graze in between meals. (#4)

Being in the study did not have a high impact. I already knew they [eating habits] were crappy and suboptimal. Maybe it will cause somewhat of a change, but not a big change. I already knew that if I wasn't getting food I would get irritable and hungry. It [initiative participation] has influenced how I would like to eat and drink but due to workplace and personal issues, I just can't make that happen. (#20)

## Discussion

Although there is a wide belief that physicians may not always attain appropriate nutrition during their work day, few empirical studies are available that help us to understand how physicians perceive the impact of inadequate nutrition upon their personal wellness and professional performance, and the barriers to achieving adequate nutrition in the workplace. Most physicians in our study believe that through improved nutrition at work they would feel better and benefit from enhanced performance, and they identified the challenges in achieving this goal. These beliefs are supported more broadly by previous research showing that physicians who suffer other workplace related strains such as stress, burnout, and sleep deprivation pose a risk both to themselves [16,17] and to the delivery of quality health care [1-9].

Nutrition is a basic wellness necessity, and although research reports some physician healthy eating behaviors [18], there is mounting evidence that physicians have difficulty accessing proper nutrition during their work day. For example, a recent cross sectional survey study by Winston found that less than half the doctors who replied reported taking regular meal breaks and that limited canteen opening times, lack of selection and lack of breaks were the most commonly perceived barriers to healthy eating while at work [11]. Examples of the importance of physicians' nutrition include a recent study demonstrating that skipping breakfast and eating meals irregularly was associated with fatigue in medical students [19] and a study linking medical students' personal nutritional habits to their patient counseling practices [20]. The physicians interviewed in our study also participated in a related nutrition based intervention study where they were fed nutritious meals and snacks at scheduled intervals during their work day. Recently published results of this research show significantly improved cognition, higher caloric intake, improved hydration, and a trend toward fewer hypoglycemic symptoms for the physicians on the intervention day compared to the day when they followed their usual workplace eating habits [15].

Our study adds to the existing literature in that the physicians interviewed not only conceded that they do not always have access to appropriate nutrition, they also aptly described their perceptions of the impact of inadequate nutrition. They portrayed the negative impact upon both their personal wellness and their ability to complete their work, recognizing that when they are undernourished, they are less likely to function at their best. In addition to identifying the practical barriers, the physicians recounted how their sense of professionalism and work ethic also hinder their work nutrition practices, a finding not previously reported to our knowledge. Lastly, the physicians in our study described how participation in a physician wellness intervention raised their awareness and altered their views of nutrition in the workplace. Unfortunately, many of the doctors admitted that the practical and professional related barriers to ensuring adequate nutrition in the workplace would still present challenges for them.

Study limitations include the relatively small sample of predominantly male physicians from a single hospital acute care setting. Also, they may have volunteered for the study because of their personal interest in nutrition and the opportunity to explore a potential association between nutrition and cognition during the wellness intervention. Future research might explore whether similar outcomes to inadequate nutrition and difficulties in accessing proper nutrition are experienced by physicians in other work settings. The interviews conducted in our study were linked to a related nutrition based wellness intervention study exploring the association between physician nutrition and cognition. The questions posed were guided by both the local knowledge of prior reporting of poor workplace nutrition and the underlying research questions: is there an association between physician nutrition and cognition, are physicians aware of this link, and are there barriers to achieving nutrition in the workplace? It is thus possible that the way in which the questions were framed may have influenced the participants' responses. However, this is not likely the case, as the participants followed up their yes/no answers and/or the subsequent probes by offering, in their own words, many and varied examples of how they personally experienced poor workplace nutrition.

## Conclusion

Physicians report that inadequate workplace nutrition has a significant negative impact on their personal wellness and professional performance. Given this threat to health care delivery, health care organizations and the medical profession need to address both the practical and professional barriers that impede physicians' basic self care issues such as nutrition, and implement physician wellness workplace initiatives that may positively influence health care organization policy, patient care, and physician behavior. Future work should include quantitative studies to determine if the major themes identified from the interviews represent those of a broader sample of physicians.

## Authors' contributions

JL contributed to the study conception and design, acquisition of data, analysis and interpretation of data, drafting and critical revision of the manuscript, obtaining funding, and administrative support. JW contributed to the study conception and design, analysis and interpretation of data, critical revision of the manuscript, and obtaining funding. KD contributed to the study design, critical revision of the manuscript and technical support. DR contributed to the conception and design of the study, critical revision of the manuscript, and technical and material support. JL had full access to all the data in the study and takes responsibility for the integrity of the data and the accuracy of the data analysis, and had final responsibility for the decision to submit the manuscript for publication. All authors read and approved the final manuscript.

## Conflict of interest statement

The authors declare that they have no competing interests.
